# T cell leukemia control via Ras-Raf pathway inhibition with peptides

**Published:** 2017

**Authors:** G Marin, H Bruzzoni-Giovanelli, G Schinella

**Affiliations:** * National University of La Plata - CONICET, Pharmacology Rebollo, Angelita; CIMI, Inserm/UPMC/CNRS- Université Pierre et Marie Curie, Paris 6, France; Centre d’Immunologie et des Maladies Infectieuses Piazzon, Isabel; National Academia of Medicine, Argentina, Immunology Researcher of the Experimental Immunology Laboratory; ** Université Paris 7- Hôpital Saint Louis, Pharmacology - Centre d’Investigations Cliniques Duarte, Alejandra; National Academia of Medicine, Experimental Immunology Laboratory; *** National University of La Plata, Pharmacology Errecalde, Jorge; Universidad Nacional de la Plata Facultad de Ciencias Medicas

**Keywords:** peptides, cancer, leukemia, control, mice

## Abstract

RATIONALE: RAS-RAF-MEK-ERK pathway has been considered a promising target for anticancer therapy. However, tumor cells may develop resistance against such drugs via hyperactivation of N-Ras, which explains why novel therapeut-ic approaches. In this sense, the Institute Curie- Université Pierre et Marie Curie (Paris 6) designed peptides in order to disturb Ras/Raf interaction which showed pro-apoptotic properties. These peptides were patented as WO2015001045 A2 (PCT/EP2014/064243)5.

OBJECTIVE: In order to check the anti-tumoral action of WO2015001045 A2 peptides in a very aggressive BALB/c mice spontaneous leukemia called LB, we performed the present study.

METHOD & RESULTS: 50 BALB/c mice inoculated with 106 LB tumor cells were randomly assigned either to control (placebo) or treatment group (that daily received 3 mg of peptide per kg of mice) during 30 days. By day 15 only 24% of the control group was alive vs. 100% of the treatment group. The average survival in treated group was 20,27 days while in control group the mean survival was 15,48 days. Either bone marrow, spleen or axillary nodes demonstrated a higher level of malignant T cell presence compare with treated group (89,78% ; 95,64% & 77,68% versus 72,45%, 80,23% & 63.44% respectively for each organ inspected.

DISCUSSION: Our study demonstrated an improvement in survival curves in mice model affected by spontaneous T lymphoid leukemia when peptides WO2015001045 A2 were used. These peptides might be a valid option to become part of the therapeutic armory for malignant lymphoproliferative diseases control.

## Introduction

Ras is a GTPase family that consists of four highly related members: K-Ras 4A, K-Ras 4B, H-Ras and N-Ras. These proteins are normally located at the inner leaflet of the plasma membrane, where they are involved in the signal transduction through interaction with multiple partners/ effectors. Ras functions as a switch between the GTP-bound active form and the GDP-bound inactive form. Active Ras binds to one of their multiple effectors, Raf serine/ threonine kinase (c-Raf or B-Raf), requires the interaction with Ras-GTP and induces their translocation to the plasma membrane where Ras is fully activated [**1**,**2**]. Ras/ Raf/ Mek/ Erk signal transduction pathway regulates cell cycle progression and apoptosis in diverse types of cells including malignant lines. This pathway can induce events associated with cell proliferation and cell cycle arrest, regulating multiple cellular processes including cell survival, growth, and differentiation. Following activation via association to GTP, Ras triggers three primary effectors, Raf, PI3K, and Ral-GDS. The three human Ras genes (H, K, and N) encode four highly related proteins. On the other hand, Raf (A, B and C) is a family of three protein serine/ threonine kinases that participate in several signaling cascades, that regulate a variety of processes like apoptosis, cell cycle progression, differentiation, proliferation and transformation to the cancerous state.

Ras mutations occur in 15-30% of all human cancers while Raf mutations may also occur in different percentages depending of the type of cancer (B-Raf mutations are present in 30-60% of the melanomas, 30-50% of the thyroid cancers, and 5-20% of the colorectal cancers) [**1**]. Certainly, the Ras and Raf mutations mentioned do not indicate all genes since these superfamilies are composed of more than 150 distinct cellular members [**2**,**3**]. 

Hence, Ras-Raf-Mek-Erk pathway has been considered a promising target for anticancer therapy [**4**,**5**]. B-Raf-inhibitors such as PLX4032 molecules are presently under investigation in clinical trials. However, there are also first hints that the tumor cells may develop resistance against such drugs via hyperactivation of N-Ras [**6**], which defines a still persisting demand for novel targeted therapeutic approaches. In this sense, Institute Curie-Université Pierre et Marie Curie (Paris 6) in France, mapped the binding sites of K-Ras to B-Raf and designed peptides in order to disturb Ras/ Raf interaction, which showed pro-apoptotic properties. These peptides were patented as WO2015001045 A2 (PCT/ EP2014/ 064243) [**7**]. Thus, the invention provides a chimeric peptide capable of cell penetration and with pro-apoptotic properties.

These peptides have demonstrated activity in several cancer lines like Lymphoma, B-Chronic Lymphoid Leukemia [**8**]. However, all these lines are based on human cancer cells transferred to mice, and because of that, immunity could have played a role in obtaining the favorable outcomes and therefore be biasing our results [**9**].

In order to avoid an immunity role of WO2015001045 A2 (PCT/ EP2014/ 064243) peptides results, we identified a non-immunogenic leukemia mice model that may confirm that the apoptotic effect of peptides is the main mechanism for anti-cancer outcomes [**9**]. This lineage is called “LB” leukemia, which is a T-lymphoid leukemia that arose spontaneously in a 6-month-old BALB/ c male. Its immune-phenotype shows CD3-, CD25+, CD8+, CD4-, gp70-, J22d.2+, MHC class I +, and MHC class II – and TCRαβ negative antigens. It is maintained by subcutaneous (s.c.) serial passages in syngeneic mice and is usually used after 80 passages. It grows to a large size, infiltrating lymph nodes, spleen, and liver [**10**]. 

LB is an extremely aggressive tumor that kills 100% of the specimens treated after 30 days of 106 cells inoculation (average latency to death, 22 days). It is enough to inject 103 viable tumor cells to give a 50% probability of lethality (LD50) [**10**]. 

To determine whether active viral replication was involved, one-month-old BALB/ c mice were inoculated intra-peritoneal with acellular extracts of LB and none of the specimens developed leukemia over a year of observation. Hence, since this leukemia is considered a non-immunogenic, non-viral induced tumor model [**9**,**10**], these features makes it ideal to develop an in vivo assay by using peptides as tools to regulate the apoptotic process; targeting proteins involved in that cell function. 

For all these reasons, LB lineage spontaneously emerged in BALB/ c mice and has become the ideal cell model to determine the true anti-tumoral action of WO2015001045 A2 (PCT/ EP2014/ 064243) peptides.

## Methods

We started an experimental procedure based on LB tumor cells intra-peritoneal (i.p.) implanted in 50 BALB/ c mice. Tumor inoculums concentrations were 106 LB cells in all cases. 100% of the specimens inoculated finally developed tumor. 

*Groups of treatment:* the 50 BALB/ c mice selected for the experience were randomly divided into two groups: Group A: “Control group” that received i.p. placebo from day 5 until day 30 and Group B: “Treatment group” that received 3 mg of peptide per kg of mice, (12 ul of peptide diluted with 138 ul of normal saline for 20 gram mice) once a day between the 5th to 30th day of the assay. During the follow up period included, peptide or placebo were daily administrated until day 30 or until the mice’ death. 

*Tumor burden anatomopathological analysis:* after the specimen was sacrificed, samples of femoral bone marrow, spleen and axillary lymph nodes were obtained. All the samples were submitted to a microscopy analysis to detect changes in tissues & cells structure and to determinate the degree of leukemia infiltration. 

*Apoptosis analysis:* These 2 specimens were removed from the initial group of 52 mice, to perform the apoptosis analysis. They were not part of the general analysis of the survival study but were injected with LB leukemia in the same manner and on the same day as the rest of the mice. One of these two specimens was left untreated while the other mouse was injected with 3mg of peptide daily from the 5th day for 5 days and then sacrificed for the apoptosis analysis in the axillary nodes, bone marrow and spleen by TUNEL technique - TMR death detection kit (Roche).

*Survival analysis:* mice status (alive/ dead) was checked twice a day. Overall survival analysis was performed at the end by Kaplan Meir method. 

## Results

All mice from the control group were dead by the 23rd day (average survival 15, 48 days). By day 15, only 24% of the group was alive, while all the mice from the intervention group were still living (**Table 1**).

**Fig. 1:**
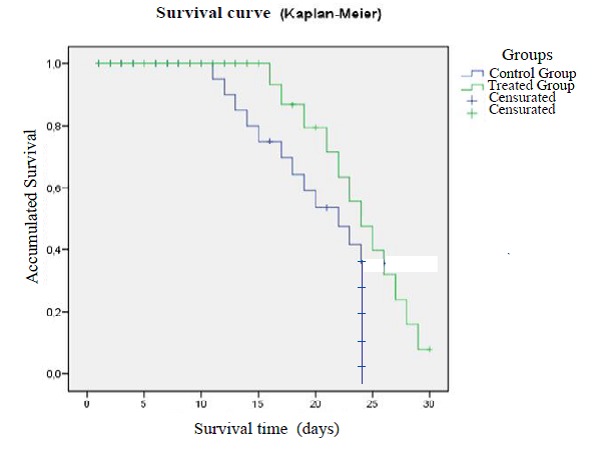
Survival analysis according to group of treatment

Although no mice in the treatment group was alive by the end of the experience (day 30), the average survival of the treated group was of 20, 27 days (range 16-28 days), while in the control group, the mean survival was of 15, 48 days (range 11-23) (**Fig. 1**).

The tumor mass estimation was performed after the specimen was sacrificed at day 30. Either bone marrow, spleen or axillary nodes demonstrated a higher level of malignant T cell presence compared to the treated group (89,78%; 95,64% & 77,68% versus 72,45%, 80,23% & 63.44% respectively, for each organ inspected).

**Table 1:** Survival analysis

		*Survival follow up*		
	Control % Survival	n (Alive)	Treated % Survival	n (Vivos)
1	100	25	100	25
2	100	25	100	25
3	100	25	100	25
4	100	25	100	25
5	100	25	100	25
6	100	25	100	25
7	100	25	100	25
8	100	25	100	25
9	100	25	100	25
10	100	25	100	25
11	96	24	100	25
12	92	23	100	25
13	88	22	100	25
14	84	21	100	25
15	76	19	100	25
16	76	19	96	24
17	72	18	92	23
18	64	16	92	23
19	52	13	88	22
20	36	9	88	22
21	36	9	84	21
22	16	4	80	20
23	8	2	72	18
24	0	0	60	15
25	0	0	52	13
26	0	0	36	9
27	0	0	8	2
28	0	0	4	1
29	0	0	0	0
30	0	0	0	0

Hence, the anatomopathological analysis demonstrated an overall tumor mass reduction in organs coming from treatment group.

**Fig. 2:**
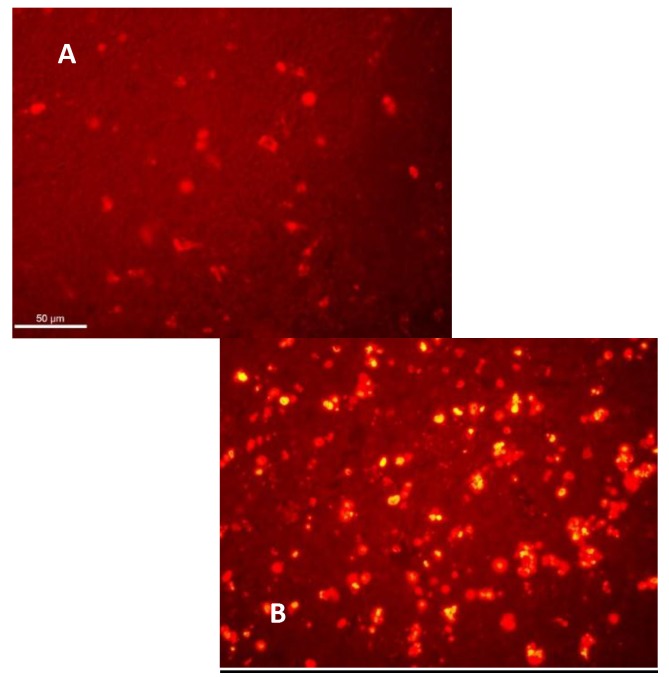
Apoptosis Analysis of tumors cells after peptide’s treatment compared to control group TUNEL + apoptotic cells in the lymph node at 48 hours of 5 days of treatment. Apoptotic (yellow) cells were observed per high-magnification 400X field in the different treatment groups.

Another observation was the different degrees of apoptosis present in most of the organs examined from the treated group, but in none of the control groups (**Fig. 2**). 

## Discussion

New anti-cancer drugs rarely demonstrate themselves highly effective, and when they do, those good results are shown by using transplantable tumors in mice. These tumor models frequently involve human tumor xenografts in mice. Unfortunately, such preclinical results are often followed by failure of the drug/ therapy in clinical trials, since most of the effects obtained were due to the immunogenicity provoked by the xenografts. As a result, a shift has occurred in scientific studies towards using spontaneous mouse tumors arising in mice, in order to avoid that reaction.

In vivo anti-tumor activity of WO2015001045 A2 (PCT/ EP2014/ 064243) peptide was demonstrated in some human tumor lines when cells were transferred to the mice model. However, the results obtained in these assays might be biased by the immunogenic murine response to a strange cell exposure. A spontaneous murine tumor like “LB” T leukemia developed in BALB/ c mice helped our team dispel these doubts. 

In this trial, we demonstrated that the peptide administration statistics improved the overall mice’ survival affected by T leukemia when compared to non-treated specimens. It also reduced the tumor mass in treated animals. Although at the very end all the mice died and the results obtained seemed to be scanty when compared to peptides inoculated to other tumors, it should be considered that LB leukemia is a very aggressive tumor that leads to the 100% death of the specimens affected, and, because of that, no treatment demonstrated efficacy so far. Besides, an improvement in the survival average rate of mice inoculated with LB T cells that received peptides (24.0 days vs. 11.5 days) had been shown. Taking into account the rapid aggressiveness after tumor injection, it could be logical to consider that peptide treatment should start before that day 5 in order to improve the results obtained even more.

## Conclusion

This study demonstrated an improvement in the survival curves in the mice model affected by spontaneous lymphoid leukemia when peptides WO2015001045 A2 (PCT/ EP2014/ 064243) are used, in order to induce apoptosis in that type of tumor. Animals treated duplicated their survival rate and showed 440 percentage of tumor reduction. Since the LB leukemia model used is a spontaneous tumor from BALB/ c mice, the possibility of immunogenicity involvement in the tumor control, is ruled out, and since the groups of mice treated were exactly obtained from the same endogamy batch of mice, the results were entirely explained by the new treatment administrated.

These peptides can be a valid option to become part of the therapeutic armory for malignant aggressive T lymphoid-proliferative diseases control. 

**Acknowledgment**

The authors would like to thank the University Group USPC in France (Université Sorbonne Paris Cité) and the National Interuniversity Council - CIN in Argentine (Consejo Interuniversitario Argentino-Antenne Universitaire Sorbonne Paris Cité) for the support in the accomplishment of the present work.
