# Correction to: T cell leukemia control via Ras-Raf pathway inhibition with peptides

**Published:** 2018

**Authors:** GH Marin, A Rebollo, H Bruzzoni-Giovanelli, G Schinella, I Piazzon, A Duarte, J Errecalde

**Affiliations:** * National University of La Plata - Pharmacology Department-CONICET; ** CIMI, Inserm/UPMC/CNRS- Université Pierre et Marie Curie, Paris 6, France; Centre d’Immunologie et des Maladies Infectieuses; *** Université Paris 7- Hôpital Saint Louis, Pharmacology - Centre d’Investigations Cliniques; **** National Academy of Medicine, Argentina, Experimental Immunology Laboratory

## Abstract

In the original publication, the authors’ names and their affiliations have been incorrectly typeset. The original article is now corrected.

In the original publication J Med Life 2017; 10:172-5 [**1**], the authors’ names and their affiliations have been incorrectly typeset. The correct author list and affiliations should read as follows: Marin GH ^1^, Rebollo A ^2^, Bruzzoni-Giovanelli H ^3^, Schinella G ^1^, Piazzon I ^4^, Duarte A ^4^, Errecalde J ^1^. ^1^ National University of La Plata - Pharmacology Department-CONICET ^2^ CIMI, Inserm/UPMC/CNRS- Université Pierre et Marie Curie, Paris 6, France; Centre d’Immunologie et des Maladies Infectieuses ^3^ Université Paris 7- Hôpital Saint Louis, Pharmacology - Centre d’Investigations Cliniques ^4^ National Academy of Medicine, Argentina, Experimental Immunology Laboratory We wish to apologise for any misunderstanding or inconvenience caused.
